# Hepatitis E virus in blood donors in England, 2016 to 2017: from selective to universal screening

**DOI:** 10.2807/1560-7917.ES.2019.24.10.1800386

**Published:** 2019-03-07

**Authors:** Heli Harvala, Patricia E Hewitt, Claire Reynolds, Callum Pearson, Becky Haywood, Kate I Tettmar, Ines Ushiro-Lumb, Susan R Brailsford, Richard Tedder, Samreen Ijaz

**Affiliations:** 1 Microbiology Services, NHS Blood and Transplant, London, United Kingdom; 2 University College London, London, United Kingdom; 3 Joint NHSBT/PHE Epidemiology Unit, Microbiology Services, NHS Blood and Transplant and Blood Safety, Hepatitis, Sexually Transmitted Infection and HIV Division, National Infections Service, Public Health England, London, United Kingdom; 4 Blood Borne Virus Unit, Virus Reference Department, Microbiology Services and National Infection Services, Public Health England, London, United Kingdom; 5 Current affiliation: Imperial College London, London, United Kingdom

**Keywords:** hepatitis E virus, blood donation, hepatitis, hepatitis E, HEV, asymptomatic, chronic, transmission, England, blood-borne infections, food-borne infections, zoonotic infections, viral infections, surveillance

## Abstract

**Introduction:**

Hepatitis E virus (HEV), the most common cause of acute hepatitis in many European countries, is transmitted through consumption of processed pork but also via blood transfusion and transplantation. HEV infection can become persistent in immunocompromised individuals.

**Aim:**

We aimed to determine the incidence and epidemiology of HEV infection in English blood donors since the introduction of donation screening in 2016.

**Methods:**

Between March 2016 and December 2017, 1,838,747 blood donations were screened for HEV RNA. Donations containing HEV RNA were further tested for serological markers, RNA quantification and viral phylogeny. Demographics, travel and diet history were analysed for all infected donors.

**Results:**

We identified 480 HEV RNA-positive blood donations during the 22-month period, most (319/480; 66%) donors were seronegative. Viral loads ranged from 1 to 3,230,000 IU/ml. All sequences belonged to genotype 3, except one which likely represents a new genotype. Most viraemic donors were over 45 years of age (279/480; 58%), donors aged between 17 and 24 years had a seven-times higher incidence of HEV infection than other donors between March and June 2016 (1:544 donations vs 1:3,830). HEV-infected blood donors were evenly distributed throughout England. Screening prevented 480 HEV RNA-positive blood donations from reaching clinical supply.

**Conclusion:**

HEV screening of blood donations is a vital step in order to provide safer blood for all recipients, but especially for the immunosuppressed. The unusually high rates of HEV infection in young blood donors may provide some insight into specific risks associated with HEV infection in England.

## Introduction

Hepatitis E virus (HEV) is a non-enveloped, single-stranded RNA virus belonging to the *Orthohepevirus* genus within the family *Hepeviridae*. Four main HEV genotypes are known to infect humans. Genotypes 1 and 2 are transmitted via the faecal-oral route between humans and cause large waterborne outbreaks in developing countries. Genotypes 3 and 4 can be transmitted to humans zoonotically from infected pigs, deer and wild boar. Transmission usually occurs through consumption of raw or inadequately cooked processed pork meat, or, rarely, by contact with infected animals or their excreta. Transmission of HEV via blood transfusion and transplantation has also been documented [1-4].

Most cases of acute HEV infections in Europe are currently caused by genotype 3 viruses [5]. Although these infections are usually asymptomatic, HEV is now recognised as the most common cause of acute viral hepatitis in many European countries including France, Germany and the United Kingdom (UK) [5]. Hepatitis E is a concern for those with underlying chronic liver disease and limited hepatic reserve as it can lead to acute-on-chronic liver failure. HEV infection offers a particular risk to persons with compromised immune systems as they may develop persistent infection which often shows a rapid progression to cirrhosis associated with a poor prognosis [6]. It has been estimated that up to two thirds of solid organ transplant recipients with persistent HEV infection develop chronic hepatitis with rapid progression of fibrosis, followed by cirrhosis and even decompensation and death [6-8]. Although there is no proven treatment for chronic HEV infection, ribavirin therapy and reduction of immunosuppression have each been successful in achieving HEV RNA clearance in individual cases [8,9].

The first systematic study investigating the potential human-to-human transmission of HEV through blood transfusion was conducted in England in 2012–2013 [4]. As part of the study, 43 recipients of HEV RNA-containing blood components were followed up; 18 had evidence of infection and a chronic infection was demonstrated in half of the HEV-infected immunosuppressed recipients [4]. Further calculations based on this study demonstrated that a minimum infective dose of 2 × 10^4^ IU is required for efficient HEV transmission by transfusion but noted that the minimum viral load in the donor plasma expected to lead to transmission was influenced by the plasma volume included in the different blood components [10]. However, transfusion risk dominates only in the heavily transfused immunosuppressed patient, whereas dietary exposure to pork-derived food has been identified as the most likely route of HEV infections in England [10-12]. As consumption of processed pork is likely to be common in the donor population, no specific donor selection criteria can be used to identify donors at enhanced risk of acquiring HEV.

In order to protect specific groups of vulnerable patients from transfusion-acquired HEV infection, blood donation screening for HEV RNA was introduced in the UK in 2016. Initially, at the end of 2015 the Department of Health and Social Care Advisory Committee on the Safety of Blood, Tissues and Organs (SaBTO) recommended the supply of HEV-screened blood components for recipients of allogeneic stem cell transplants and solid organ transplants [13]. In March 2016, NHS Blood and Transplant (NHSBT) introduced HEV NAT screening on pools of 24 for selected blood donations in England. It was anticipated that a minimum of 30% of the blood supply would need to be tested in order to meet clinical demand, including screening of all platelets donated by apheresis. Donations identified as HEV RNA-positive were excluded from the supply; whole blood donors were suspended for 6 months from the date of their HEV RNA-positive donation whereas apheresis platelet donors were re-instated once they had cleared the infection and developed a high concentration of antibody to HEV (anti-HEV IgG, sample/cutoff (S/CO) > 10).

A change to universal screening of donations in April 2017 followed a further review by SaBTO, which considered that HEV-screened blood should also be supplied for all immunocompromised patients [14]. It was also estimated to be a cost-neutral change, provided that the incidence of HEV infection remained above 1 in 10,000 blood donations. Universal screening of blood for HEV RNA was thought to be as beneficial for patients as selective screening but was estimated to provide an easier and more economical workflow in both hospitals and screening laboratories. In hospitals it would reduce the need for two separate inventories for blood, with a lower risk of errors occurring during the allocation process.

Here we present the results of routine screening of almost two million blood donations for HEV RNA between March 2016 and December 2017 in England (n = 1,838,747). We aimed to determine the incidence of HEV among English blood donors, and to describe the classical and phylogenetic epidemiology of HEV infections in these donors.

## Methods

### Detection and characterisation of donor samples

All apheresis and selected whole blood donations collected in England were tested for HEV RNA from 1 March 2016 to 9 April 2017; universal blood donation screening was introduced on 10 April 2017. Up to 31 December 2017, 1,838,747 blood donations were screened for HEV RNA (selective screening: 662,162 donations; universal screening: 1,176,585 donations). Minipools of 24 donations were assembled and screened for HEV RNA with an internally controlled RT-PCR with reported 95% of limit of detection of 18.6 IU/ml (Cobas, Roche, Burgess Hill, UK) and calculated 95% of limit of detection 446 IU/ml in individual donor level when tested in pools of 24. Reactive pools were resolved to individual HEV RNA containing donations using the same assay, and individual samples were subjected to further testing at the National Transfusion Microbiology Reference Laboratory (NHSBT, Colindale, UK). Nucleic acid was extracted from individual samples using the EZ1 Advanced XL system (Qiagen, Crawley, UK) and amplified using either an in-house real-time PCR for HEV RNA detection from March 2016 to April 2017 or from May to September 2017 using the ampliCube HEV 2.0 assay (Mikrogen Diagnostik, Neuried, Germany) with a reported 95% limit of detection of 36.13 IU/ml. From October 2017 onwards, the presence of HEV RNA in individual samples was confirmed using the Procleix Panther HEV assay with a reported 95% limit of detection of 7.9 IU/ml (Grifols Diagnostic Solutions Inc; developed in collaboration with Hologic Inc, Cambridge, UK). HEV serology was performed using the Wantai immunoglobulin IgM and IgG detection assays (Fortress Diagnostics, Antrim, Northern Ireland). HEV RNA quantification and virus sequencing of a 1,115-nt-long region across the open reading frame 2 (ORF-2) were performed at the Blood Borne Virus Unit, Public Health England (PHE), Colindale, UK, as previously described [15]. Phylogenetic analysis was performed using MEGA6 [16].

### Notification and follow-up of hepatitis E virus-infected donors

All viraemic donors were sent a notification letter explaining their test results and an information leaflet about HEV infection. They were given an opportunity to telephone and discuss their test results with a member of the NHSBT clinical team. Details of the donors’ HEV infections were also sent to their general practitioners with the donors’ consent and to the local health protection teams. Apheresis platelet donors were informed that they would not be able to donate again until further blood sampling confirmed viral clearance; a follow-up sample was requested from all apheresis donors within 6–8 weeks from diagnosis, whereas whole blood donors were asked to wait for 6 months before returning to donate.

Donor details, including age, sex and postcode, were collected for all HEV RNA-positive donors. R-studio (Free software foundation, established in Auckland, New Zealand) was used to match postcode by PHE region, to be then mapped using ArcGIS (ESRI, Aylesbury, UK) to display viraemic donors.

### Archived sample

The archived plasma sample from the most recent negative donation was retrieved for all RNA-positive apheresis donations and tested for HEV RNA and antibodies. Archive samples were available for 35 of 45 HEV-infected apheresis donors. If the most recent archive was identified to contain HEV RNA, the next most recent archive sample was also retrieved for testing. Hospitals which had received the HEV RNA-containing blood components were informed of potential risks and advised to take appropriate action.

### Questionnaire

The NHSBT clinical team completed a questionnaire for each confirmed viraemic donor for surveillance purposes. Donors were also given an opportunity to call back and discuss their results with a clinician. This discussion included questions about travel 6 weeks prior to donation, food history (meat, meat excluding pork or vegetarian) as well as signs and symptoms experienced around the time of donation.

### Ethical statement

Signed consent was obtained from each donor at the time of donation. Donors consent to NHSBT holding information about them including their health, attendances and donations and using their information for the purposes explained in the donor welcome booklet and data protection leaflet which donors are asked to read at the time of donation. This includes using data for the purposes of clinical audit to assess and improve the service and for research, specifically to improve our knowledge of the donor population.

### Statistical analysis

Categorical variables were compared using chi-squared tests in VassarStat (Poughkeepsie, NY, US), and geometric mean viral loads were calculated and further compared using Kruskall-Wallace in Systat version 10.2 (Systat Software Inc., San Jose, California, US).

## Results

### Blood donation screening for hepatitis E virus

**Figure 1:**
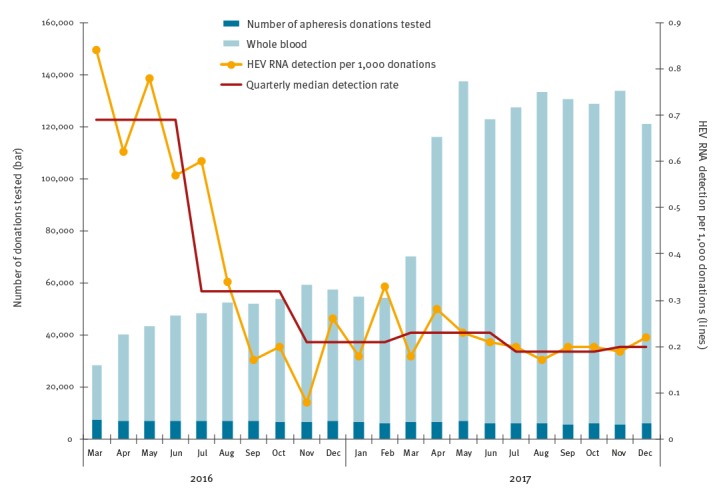
Number of apheresis platelet (n = 148,439) and whole blood donations (n = 1,609,308) tested, and hepatitis E virus RNA detection rate per 1,000 donations (n = 480) by month, England, 1 March 2016−31 December 2017

A total of 662,162 donations (569,669 whole blood and 92,493 apheresis donations) were screened for HEV RNA between 1 March 2016 and 9 April 2017; 239 donations were identified as positive (Figure 1). A further 241 donations were identified as HEV RNA-positive from 1,176,585 screened between 10 April and 31 December 2017. All reactive pools were resolved to a single reactive donation. The number of donations screened for HEV RNA per month increased from an average of 50,931 (range: 28,491–70,303) during selective screening to 127,953 (range: 116,066–137,372) when universal screening was introduced. At the same time, the detection rate decreased from 0.69 per 1,000 donations at the start of the selective screening period (March to June 2016) to 0.32 per 1,000 donations in the middle of the selective screening period (July to October 2016), and to 0.21 in the end of the selective screening period (November 2016 to February 2017). The detection rate subsequently stayed steady at around 0.2 per 1,000 donations (March to December 2017). The method used for screening of donations in pools of 24 did not change during this time.

HEV: hepatitis E virus.

Quarterly median detection rates per 1,000 donations have also been calculated.

### Virology

**Figure 2:**
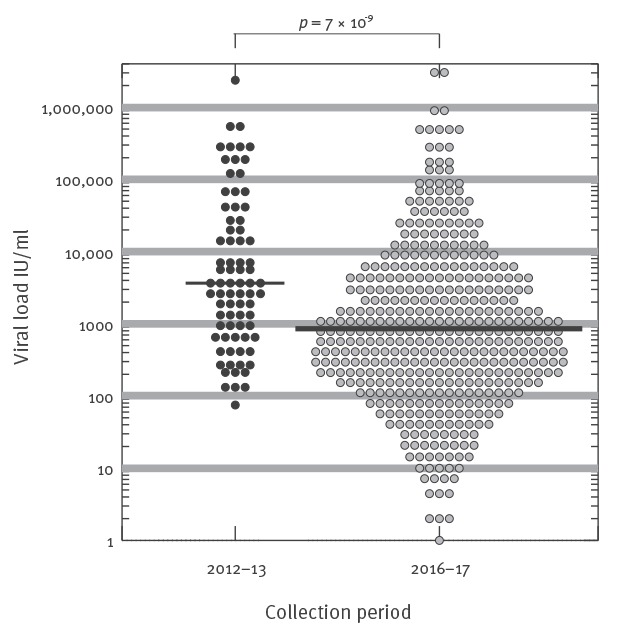
Hepatitis E virus viral loads in individual blood donors, England, 2012–2013 and 2016–2017 (n = 406)

**Figure 3:**
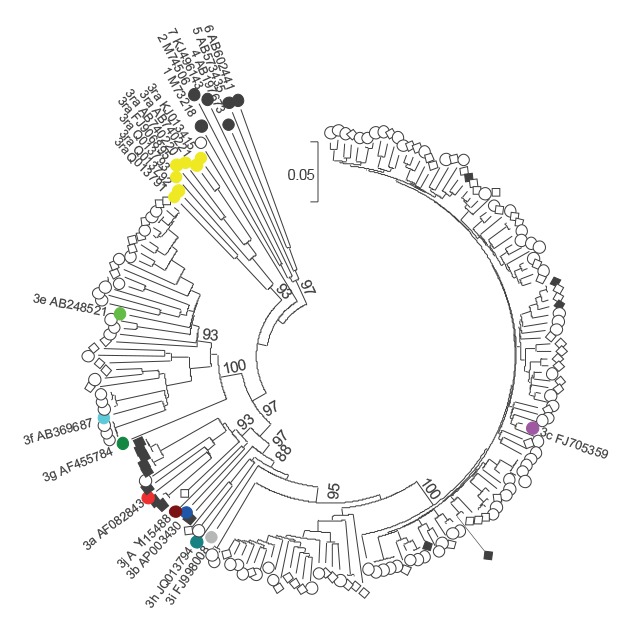
Phylogenetic analysis of hepatitis E virus variants from blood donors, England, March 2016−December 2017 (n = 150)

The geometric mean viral load was 883 IU/ml (range: 1–3,230,000 IU/ml), which was significantly lower than detected in English blood donors in a previous study (Figure 2 [4]). Of the 480 samples, 150 were successfully genotyped (31%), and all except one sequence belonged to genotype 3 (Figure 3). The exception likely represents a new HEV genotype. The majority of virus strains clustered with HEV subgenotype 3c (112/149), whereas most of the remaining 34 sequences clustered either with 3e (21/149), 3f (12/149) or 3a (1/149). Interestingly, two further sequences were only distantly related to 3h, and one sequence clustered distantly with 3a.

Geometric mean viral load was significantly higher in the 2012–13 testing period than in 2016–17 (4,740 IU/ml vs 883 IU/ml, p = 7 × 10^–9^). Viral load data for 2012–13 has already been published [4].

MEGA 6.0 software was used to construct the maximum composite likelihood tree. Bootstrap values >85% are indicated in the branch nodes. The 1,115 nt-long ORF2 sequences (nt 5792–6907, numbered relative to M73218) were obtained from blood donors in 2016 (open diamond) or 2017 (open circle); sequences obtained from donors under the age of 24 years between March and July 2016 (black diamond) were compared with reference sequences of HEV-3 subgenotypes (coloured) and other HEV genotypes (black circles), as well as with HEV strains isolated from rabbits (yellow circles) or pigs (black squares).

Two thirds of samples from viraemic donors (319/480, 66%) were unreactive for anti-HEV IgG and IgM at the time of donation), whereas 103 samples (21%) were reactive for both anti-HEV IgG and IgM, 42 (9%) were reactive only for anti-HEV IgG antibodies and 16 (3%) were reactive for anti-HEV IgM alone. Follow-up samples were received from 21 apheresis donors between 1 March 2016 and 31 March 2017; in all cases the donor developed an anti-HEV IgG response, seven without concurrent anti-HEV IgM antibodies at the time of sampling.

An immediate previously negative or non-tested archived sample was available for 35 of 45 apheresis donors; three of these archived samples were found to contain HEV RNA with viral loads of 4 (donation tested negative on 30 April 2016), 8 (donation tested negative on 20 March 2016) and 3,476 IU/ml (no donation taken; appointment on 30 May 2016 for sample only, due to a recent acupuncture, and sample not screened for HEV RNA). The next most recently archived sample was available for one of these donors and found not to contain HEV RNA.

### Donor demography

**Figure 4:**
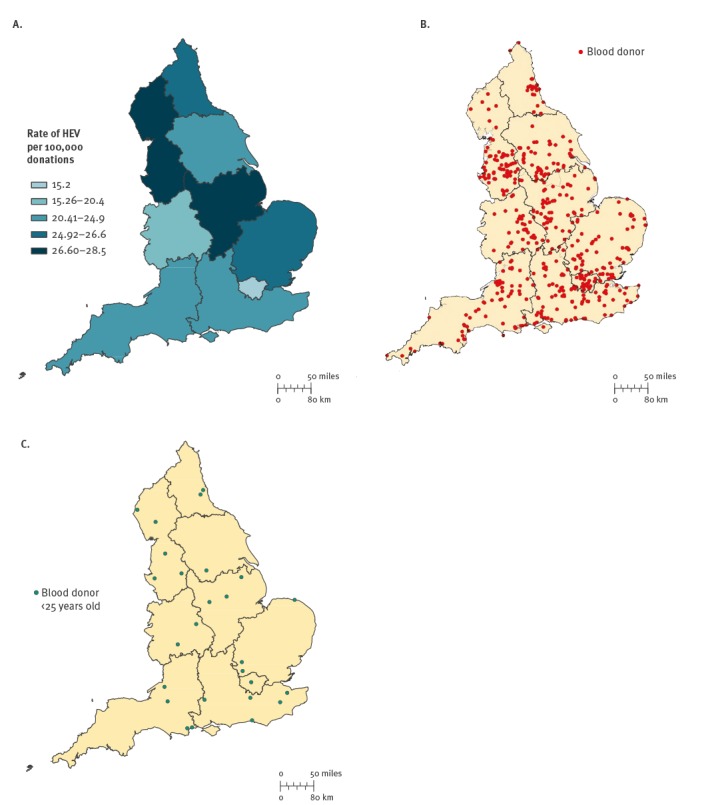
(A) Hepatitis E virus RNA detection rate per 100,000 blood donations, England, March 2016−December 2017 (n = 480); (B) Geospatial location of hepatitis E virus-infected blood donors, England, March 2016−December 2017 (n = 480) and (C) Geospatial location of hepatitis E virus-infected blood donors under the age of 25 years, England, March 2016−July 2016 (n = 26)

Between March 2016 and December 2017, HEV-infected blood donors did not appear to cluster in England (Figure 4). Twenty six cases were identified in the London area. The remaining 454 cases were evenly spread between the south of England (n = 158), the middle of England (n = 157) and the north of England (n = 139). The prevalence of HEV RNA per 100,000 blood donations varied from 15.2 in the London area to 20.4–27.5 elsewhere in England.

HEV: hepatitis E virus.

**Figure 5:**
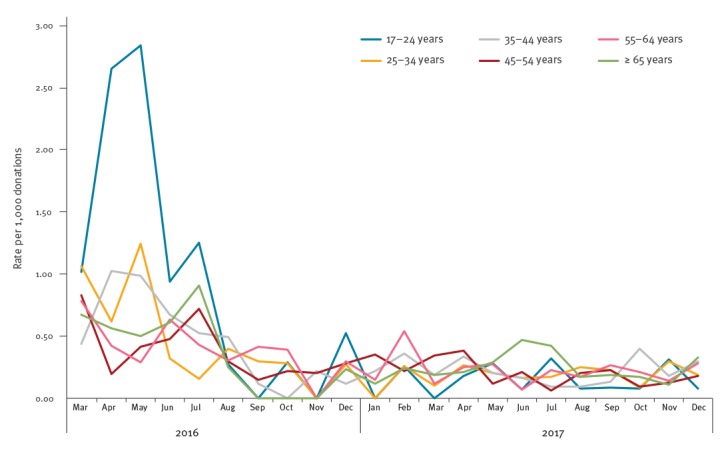
Rate of hepatitis E virus RNA-positive donations by age groups, England, March 2016−December 2017

A total of 435 HEV RNA-positive donations were from 1,690,308 whole blood donations screened, and the remaining 45 were from 148,439 apheresis donations. Over 90% of HEV-positive donations were from repeat blood donors (455/480). Most donors reported their ethnic group as white British or white other (n = 461), whereas ethnic group was non-white for six donors and not known for 13. There was a significantly higher proportion of males infected than females (313/975,953 and 167/862,794; p < 0.0001). Although HEV RNA was seen in all donor age groups, the highest rate seen in any age group was that seen in 17–24-year-old donors between March and July 2016 (Figure 5). This normalised after July 2016 to the level recorded in other age groups.

### Donor risk factors for hepatitis E virus infection

Travel history was available for 355 donors, and 82 (23%) reported travel outside the UK in the 6 weeks before donation. Information on diet was available for 348 donors; most HEV-infected blood donors had consumed meat including pork (339/348; 97%). Seven blood donors had eaten meat but not pork; one reported eating pâté, one had swum in water assumed to be contaminated, one lived near a pig farm in England, one had consumed shellfish and one had recently travelled to Croatia. The remaining two donors were vegetarians; one of them worked at a garden centre in England that used manure/compost and the other ate pre-packed salads.

### Self-reported clinical signs and symptoms

A total of 334 (70%) HEV RNA viraemic donors provided information on clinical signs and symptoms and 146 reported one or more (Table). Fatigue was the most commonly reported symptom after donation (n = 85), but some donors also reported joint pain/aches (n = 22). A small number of donors reported jaundice (n = 6) after donation.

**Table:** Clinical signs or symptoms experienced by blood donors before, around or after donation based on self-reporting, England, March 2016−December 2017 (n = 146)

Symptom or clinical sign^a^	Before donation	Around donation	After donation
Fatigue	37	38	85
Joint pain/aches	9	4	22
Feeling ill	11	1	21
Nausea	7	1	20
Change in appetite	5	1	10
Abdominal pain	6	2	13
Fever	5	3	7
Vomiting	4	0	5
Dark urine	5	5	18
Jaundice	1	1	6

^a^ Donors could report multiple signs and symptoms, and at multiple time points.

## Discussion

The detection of HEV RNA in 480 of 1,838,747 blood donations demonstrates a high incidence of HEV infection in England, accounting for an overall prevalence of 1 in 3,830 donations during the period between March 2016 and December 2017. A similar prevalence of viraemia in blood donors has been previously reported in many other European countries, indicating the wide spread of this infection in European human populations [17]. However, over the years it has become noticeable that the rate of HEV RNA-containing donations fluctuates considerably over time. A prevalence of 1 in 2,848 donations was noted in a previous study from England in 2012–13 [4], whereas a higher prevalence of 1 in 1,365 was reported 3 months after selective screening was introduced throughout England in March 2016 ([17]; Figure 1). Interestingly, the detection rate has decreased since then, and has remained steady around 0.20 per 1,000 donations (1/4,781 donations) in England. However, we have not changed the method used for screening of donation in pools of 24 during this time period, and if anything, it seems to have become more sensitive. It remains to be seen how the prevalence of HEV RNA viraemic blood donors will change in the future.

More than half of HEV-infected blood donors were over 45 years old (279/480), which is in line with previously published data [18,19]. However, younger donors aged 17­–24 years were shown to have a seven-times higher risk of acquiring HEV infection when compared with other individuals who had donated between March and July 2016 (1:544 donations vs 1:3,830, Figure 5). This normalised after July 2016 to the level recorded in other age groups. Interestingly, this was also mirrored in the English HEV surveillance programme where data on laboratory-confirmed cases of HEV infections are collected from reference and local laboratories [20]; 20% of all infections were reported to be in 15–24-year-old individuals between April and September 2016 (94/471) whereas only 4% of HEV infections were seen in this age group between October 2016 and December 2017 (43/1,088). These young donors were distributed throughout England, and they were infected with a variety of HEV 3c subgenotype strains (Figures 3 and 5). In the future, we will aim to analyse our incidence data across the different age groups in order to identify possible ongoing outbreaks and investigate and mitigate possible sources of infection.

As in many previous studies, we identified a dietary exposure to pork-derived foods for most HEV-infected donors. It is interesting that the rate of HEV RNA detection was lowest in the London area (15.2/100,000 donations; Figure 4) with rates elsewhere (of 20.4–26.2/100,000 donations). Whether this could reflect the over-representation of vegetarian donors living in the London area remains to be studied further. Six donors (1.3%) reported jaundice after donation. Although it is difficult to verify these reports, the figure is in keeping with the previous study, where one of 79 HEV RNA-positive blood donors developed mild post-donation hepatitis [4].

Our study also demonstrates the diversity of HEV strains circulating in England, and that we identified a potentially new genotype and two new subgenotypes. However, a whole genome sequence is required for formal assignment of new HEV genotypes. Based on the phylogenetic analysis of 150 HEV infected blood donors, all except one infection were caused by HEV genotype 3 and showed the dominance of subtype 3c variants within the clade-2/abjchi (110/150, 73%; Figure 3). A proposed new genotype was identified from a blood donor with previous travel to France, but without any specific dietary exposures. The replacement of clade-1/efg by these clade 2/abjchi variants was already noted in England in 2011, and the predominance of subgenotype c (33/54, 61%) has been obvious since 2012–2013 [4,21,22]. Genotype 3 viruses are known to transmit zoonotically, with pigs, wild boar and deer acting as a reservoir for human infections. It is clear that the majority of these infections were acquired in England, as 75% of donors reported no travel abroad in the 6 weeks before donation. Although there are limited sequence data available from English pigs harbouring HEV, it is thought that clade-2/abjchi viruses found in pigs in several European countries are rare among indigenous UK pigs [23]. HEV subgenotype 3c variant was identified only in one caecal content sample, whereas sequences from 22 other samples fall into clade-1/efg. Furthermore, it has been suggested that human infections with genotype 3 clade-2/abjchi variants in England may be largely due to consumption of food products made from pork originating outside the UK [24]. However, further data are needed to understand the circulation of HEV in the UK pig population and the source of infection in blood donors.

Although selective HEV RNA screening was initially implemented in England, a change to universal screening of donations in April 2017 was driven by its comparable costs and easier logistical performance. In addition to the UK, universal HEV RNA screening of blood donations has been implemented in Ireland [25], the Netherlands [17], six German donor centres [17] and parts of Japan [26]. Other countries are either still investigating or considering whether blood donation screening for HEV RNA is needed [17]. Denmark has deemed HEV screening unnecessary as a low prevalence of viraemic donors was detected and no evidence of transfusion-transmitted infections has been found [27]. Similarly, donor screening for HEV RNA has not been introduced in Australia due to the low prevalence estimates of 1 in 74,313 [28].

We identified a total of 480 HEV RNA-positive blood donors over the 22-month screening period, who presented with median viral load of 883 IU/ml. This was significantly lower than previously detected in English blood donors in 2012–13, the slightly increased reported sensitivity of the testing assays used in between 2016 and 2017 (15%) is unlikely to account for this difference [4]. Large numbers of donors were identified with a very low viral load below the stated sensitivity of the screening assay used. To investigate this further, parallel comparison of screening and confirmatory assays used for HEV testing should be performed using the dilution series of the World Health Organization HEV standards. A total of 42 of 480 HEV RNA-positive donors were reactive for anti-HEV IgG antibodies only (no IgM) at the time of donation, reflecting either late infections or possible re-infection.

No new cases of transfusion-transmitted (TT) HEV were reported in England during the study period. Interestingly, we have only detected two donations missed by initial pooled screening, both with very low viral load (4 and 8 IU/ml). A previous study estimated that around 55% of recipients challenged with a component containing a minimum of 20,000 IU of HEV RNA would become infected [10]; this would mean that a viral load of 1,600 IU/ml in a donor (whole blood donation) and 111 IU/ml (apheresis platelet donor) would be sufficient to transmit infection to recipients, in keeping with previous observational studies [4,29]. Based on the number of donations where HEV RNA viral load exceeded these cut-offs, it can be speculated that with universal HEV screening a total of 146 donations with the potential to transmit HEV infection with severe outcomes particularly in immunocompromised transplant recipients, patients with haematological malignancies and underlying liver disease, have been removed from clinical use.

As well as mitigating the risk of transfusion acquired HEV, the screening of blood donations provides a useful and unique insight into HEV infections at a population level. The data obtained inform on fluctuations in risk and changes in HEV incidence and allow for comment to be made on what is largely an asymptomatic infection. They are essential to the development of guidelines for public health purposes and to inform continued risk assessments around blood safety.
